# Beyond viral suppression: decoding the mitochondrial-immune axis in HIV-associated inflammation and immune dysfunction

**DOI:** 10.3389/fcimb.2025.1686785

**Published:** 2025-10-16

**Authors:** Tracy Okine, Eleanor Hill, Kate Sheran, Talia H. Swartz

**Affiliations:** Department of Medicine, Division of Infectious Diseases, Icahn School of Medicine at Mount Sinai, New York, NY, United States

**Keywords:** HIV, mitochondria, inflammation, OxPhos, inflammasome, ATP

## Abstract

Antiretroviral therapy (ART) has transformed HIV into a chronic, manageable condition, yet people living with HIV (PLWH) continue to experience persistent immune activation and systemic inflammation that drive long-term comorbidities, including neurocognitive impairment and cardiovascular disease. This residual inflammation requires new mechanistic explanations and targeted therapeutic approaches. Increasing evidence highlights mitochondria as central hubs in the regulation of cellular metabolism and immune responses. In PLWH, both HIV and ART disrupt mitochondrial function, leading to the release of proinflammatory mediators such as reactive oxygen species (ROS) and oxidized mitochondrial DNA (mtDNA). These signals activate the NLRP3 inflammasome, resulting in secretion of IL-1β and other cytokines. In parallel, excess mitochondrial ATP engages purinergic receptors such as P2X1 and P2X7, propagating inflammatory signaling to surrounding immune cells. This review examines the mito–immune axis in HIV, focusing on OxPhos dysregulation, inflammasome activation, and purinergic receptor signaling, and explores potential interventions—including purinergic antagonists—that aim not only to suppress viral replication but also to restore immunometabolic balance. By recognizing mitochondria as dynamic regulators of immune function, we outline a paradigm shift in HIV treatment that addresses the underlying drivers of chronic inflammation.

## 1 Introduction

HIV infection results in progressive immune dysfunction characterized by chronic inflammation, immune activation, and gradual loss of CD4^+^ T cells (Deeks et al., 2013; Ellis et al., 2021). If untreated, this trajectory culminates in acquired immunodeficiency syndrome (AIDS), with profound susceptibility to opportunistic infections. ART has dramatically altered this natural history, effectively suppressing viral replication and reducing AIDS-related mortality (Maagaard and Kvale, 2009; McComsey et al., 2013). However, even among individuals with durable viral suppression, residual immune activation and systemic inflammation persist (Deeks et al., 2013; Ellis et al., 2021). These abnormalities are influenced by ongoing low-level viral activity, the legacy of immune damage, co-infections, microbial translocation, and ART-associated toxicity (Feeney et al., 2012; Mikaeloff et al., 2023). Early studies demonstrated that massive depletion of CD4^+^ T cells in the gastrointestinal tract drives epithelial barrier dysfunction and microbial translocation, which in turn fuel chronic immune activation (Brenchley et al., 2004).

A growing body of evidence suggests that mitochondrial dysfunction plays a crucial role in contributing to this inflammatory environment (Valle-Casuso et al., 2019; Kallianpur et al., 2020; Freeman et al., 2023). Once regarded purely as energy-generating organelles, mitochondria are now understood to act as integrated platforms for metabolic and immune signaling (Patrick and Watson, 2021). In the context of HIV and ART, mitochondrial dysregulation can drive chronic inflammation through multiple mechanisms, including disruption of OxPhos, release of mitochondrial danger signals, and altered immune cell metabolism. These processes are linked to long-term complications such as neurocognitive decline, gut barrier impairment, and cardiovascular disease (Kallianpur et al., 2020; Ambikan et al., 2022). Defining the mechanisms by which the mito–immune axis is perturbed in HIV may enable the development of targeted interventions that address comorbidity risk in PLWH. Modern HIV treatment relies on combination regimens, most commonly integrase strand transfer inhibitors (INSTIs) combined with nucleoside reverse transcriptase inhibitors (NRTIs). Historically, protease inhibitor (PI)–based regimens and older NRTIs such as zidovudine and stavudine were widely used but carried greater mitochondrial toxicity, leading to metabolic complications (Maagaard and Kvale, 2009; Feeney et al., 2012; McComsey et al., 2013; Rodriguez et al., 2024). The evolution of these therapeutic backbones provides important context for interpreting how ART intersects with HIV-driven metabolic remodeling.

## 2 OxPhos reprogramming and dysfunction in HIV

OxPhos is a central mitochondrial pathway for ATP production via the electron transport chain (ETC). In HIV infection, OxPhos is reprogrammed. Infected cells, particularly activated CD4^+^ T cells, show a consistent shift toward glycolysis and fatty acid oxidation, accompanied by mitochondrial hyperactivation and accumulation of reactive oxygen species (ROS), as demonstrated in ex vivo human lymphoid tissue and animal models (Valle-Casuso et al., 2019; Freeman et al., 2023; Rodriguez et al., 2024). This reprogramming may fulfill the elevated biosynthetic and energetic demands of viral replication but also predisposes mitochondria to stress responses that release ROS and ATP—two upstream triggers of NLRP3 inflammasome activation (Ekabe et al., 2021; Guo et al., 2021; Freeman et al., 2023). While the glycolytic shift is well-documented, the relative contribution of OxPhos upregulation versus functional impairment likely varies by cell type, stage of infection, and ART exposure, underscoring the need for longitudinal, cell-specific studies in PLWH. HIV also induces transcriptional changes in ETC components, particularly Complex IV, which reduce the efficiency of ATP synthesis (Tripathy and Mitra, 2010; Kallianpur et al., 2020). These disruptions contribute to cellular stress and further sensitize immune cells to inflammasome activation and functional dysregulation.

Beyond altering cellular energy production, HIV-associated OxPhos reprogramming modifies mitochondrial membrane potential, ROS signaling thresholds, and metabolite profiles such as succinate and citrate—changes that influence inflammasome priming and cytokine production (Kepp et al., 2011; Hileman et al., 2021). These bioenergetic alterations may also lower the mitochondrial reserve capacity, potentially increasing susceptibility to secondary insults. In PLWH, one such secondary factor is ART exposure, which can impose distinct, regimen-specific mitochondrial stresses. The combined effects of viral-induced metabolic remodeling and ART-associated toxicity can interact to amplify oxidative stress, disrupt mtDNA maintenance, and accelerate mitochondrial aging, as discussed in the following section.

## 3 Antiretroviral therapy–associated effects on mitochondrial function

While ART has transformed HIV into a chronic, manageable condition, its effects on mitochondrial health are regimen-dependent. Older nucleoside reverse transcriptase inhibitors (NRTIs), such as zidovudine and stavudine, directly inhibit mitochondrial DNA polymerase γ, leading to profound depletion of mtDNA, reduced synthesis of OxPhos proteins, and impaired respiratory chain activity. Protease inhibitors (PIs) have been associated with altered mitochondrial lipid metabolism and additional oxidative stress, contributing to metabolic complications such as lactic acidosis and lipodystrophy (Maagaard and Kvale, 2009; Feeney et al., 2012; McComsey et al., 2013; Mikaeloff et al., 2023). Although modern integrase inhibitor–based regimens have fewer overt mitochondrial toxicities, emerging evidence indicates that subtle, chronic effects persist. These may include modest reductions in mtDNA copy number, increased markers of oxidative damage, and altered mitochondrial dynamics, even in the absence of clinical symptoms. Importantly, these ART-related changes can occur on the background of HIV-driven metabolic remodeling, creating additive or synergistic stress on mitochondrial function.

By impairing ATP generation, increasing ROS production, and promoting mtDNA instability, ART can exacerbate the same pathways activated by HIV infection, including those that prime the NLRP3 inflammasome. Understanding how specific ART components influence mitochondrial bioenergetics is essential for optimizing therapy in PLWH, particularly those at increased risk for inflammation-associated comorbidities.

### 3.1 Impact on immune cell metabolism

HIV-driven mitochondrial dysfunction also reshapes the metabolic landscape of key immune cells. CD4+ and CD8+ T cells, as well as macrophages, undergo metabolic reprogramming during HIV infection (Castellano et al., 2019; Valle-Casuso et al., 2019; Alrubayyi et al., 2022; Mikaeloff et al., 2023). For instance, T cells exhibit impaired OxPhos and increased dependence on glycolysis, a shift associated with immune exhaustion and diminished antiviral responses. This metabolic remodeling has functional consequences. Disruption of OxPhos contributes to the loss of effector function, impaired immune surveillance, and sustained inflammation, all of which are hallmarks of chronic HIV infection (Guo et al., 2021; Ambikan et al., 2022). By altering how immune cells generate energy and respond to stress, mitochondrial dysfunction becomes a key driver of HIV pathogenesis. Together, these insights position the mito-immune axis as a critical node in understanding the persistent inflammation seen in HIV. Focusing on this axis, particularly mechanisms like NLRP3 inflammasome activation, may reveal new therapeutic targets that extend beyond viral suppression.

### 3.2 NLRP3 inflammasome and HIV

The NLRP3 inflammasome is a critical node linking mitochondrial stress to downstream inflammation in HIV infection. Human tissue studies confirm elevated NLRP3 components and IL-1β in PLWH, but direct causal evidence connecting NLRP3 activation to specific comorbidities such as CVD remains limited. Much of the cardiovascular link is inferred from biomarker associations (IL-6, hsCRP, D-dimer) and animal models in which NLRP3 inhibition reduces atherosclerotic burden. Consequently, while NLRP3 is a promising therapeutic target, its exact contribution to disease progression in treated PLWH is still being defined.

These cytosolic proteins activate caspases that regulate inflammation and apoptosis (Ekabe et al., 2021). The NLRP3 inflammasome mediates caspase-1 activation and the secretion of proinflammatory cytokines IL-1β and IL-18 (Kelley et al., 2019). During HIV infection, NLRP3 activation contributes to neuroinflammation in the central nervous system (CNS) and promotes CD4+ T cell apoptosis (Doitsh et al., 2010; Feria et al., 2018). Rodent models of HIV associated neurocognitive disorders (HAND), such as Alzheimer’s disease, bipolar disorder, and Parkinson’s, further display NLRP3’s role in brain dysfunction (Torices et al., 2023). In Parkinson’s, elevated IL-1β and CNS protein inclusions in the gut indicate NLRP3 overactivation (Pellegrini et al., 2020). Enteric bacteria may further activate NLRP3, influencing peripheral nervous system (PNS) and CNS responses. NLRP3-related inflammation is also linked to cardiovascular disease (CVD) and atherosclerosis in PLWH on antiretroviral therapy (ART). This risk is associated with elevated baseline levels of IL-6, hsCRP, and D-dimer, all indicators of bodily inflammation in PLWH using ART (Duprez et al., 2012). NLRP3 blockage may reduce IL-1β activity and heart disease progression, suggesting a correlation between CVD and HIV infection (Duprez et al., 2012; Bracey et al., 2013). These findings underscore NLRP3’s role in linking HIV-induced immune activation with systemic comorbidities, positioning it as a promising therapeutic target.

Reactive oxygen species (ROS) and oxidized mtDNA are critical modulators of NLRP3 inflammasome activation, linking cellular stress to HIV-related inflammation. ROS are byproducts of mitochondrial metabolism and activate NLRP3. ROS from complexes I and III of the electron transport chain (ETC) stimulate IL-1β release and interact with kinases such as mitogen-activated protein kinase (MAPK) and extracellular signal-related kinases 1 and 2 (ERK1/2) (Kepp et al., 2011). MAPK is a kinase crucial for transducing signals from the outside of the mitochondria to the inside, including mediating cytokines such as TNF-alpha and IL-10, increasing ROS production, and decreasing the activity of antioxidant enzymes (Dhingra et al., 2007). This results in increased oxidative stress, modulated by ERK1/2, which directs the mitochondria to either a pro- or anti-inflammatory state (Dhingra et al., 2007). These findings highlight how mitochondrial dysfunction not only amplifies inflammation via the NLRP3 pathway but also significantly contributes to HIV-associated immune activation and neuroinflammation.

Notably, ROS can have both activating and inhibitory effects. Though ROS can directly activate caspase-1 and indirectly activate caspase-3 via ERK1/2 (Cruz et al., 2007), excessive ROS can suppress IL-1β secretion, as seen in SOD-1-deficient macrophages (van de Veerdonk et al., 2010). This paradox is explained by oxidative damage decreasing redox-sensitive molecules, such as caspase-1 (Meissner et al., 2008). Thus, while ROS are key players in NLRP3 activation, their effects are context-dependent and oscillate between inflammation and redox activation. In addition to ROS, other mitochondrial byproducts such as oxidized mitochondrial DNA (mtDNA) serve as potent activators of NLRP3 under conditions of cellular stress. Oxidized mtDNA acts as a ligand for NLRP3 and is critical for inflammasome signaling (Kim et al., 2023). Altogether, the dual role of ROS and proinflammatory signaling highlights how mitochondrial stress is underscored by oxidized mtDNA as a nuanced and dynamic indicator of inflammasome activation.

### 3.3 Crosstalk between mitochondria and inflammasome

HIV proteins, including Tat, Vpr, and gp120 facilitate communication between the mitochondria and the inflammasome (Jones et al., 2007; Chivero et al., 2017; Arjona et al., 2023). Tat, or the transactivator of transcription, enhances IL-1β secretion, activates NLRP3 in microglia, and disrupts mitochondrial integrity through PTPIP5-mediated ROS accumulation (Chivero et al., 2017; Arjona et al., 2023). Viral protein R (Vpr) promotes the expression of neuroinflammatory markers and neuronal apoptosis signaling through the activation of caspases and the release of cytochrome c (Jones et al., 2007; Williams et al., 2023). Gp120, which mediates viral entry, also contributes to immune dysregulation and the internalization of CD4+ T cells (Yoon et al., 2010). Crosstalk is also essential for maintaining autophagy, a process that clears damaged mitochondria, which HIV disrupts to create an ideal environment for viral replication (Sun et al., 2024). Beyond autophagy, mitochondria-associated membranes (MAMs) serve as an interface between the endoplasmic reticulum (ER) and mitochondria, facilitating communication. MAM disruption contributes to cognitive decline in PLWH (Arjona et al., 2023). These mitochondrial-ER contact sites not only influence immune signaling but are part of a network of mitochondrial maintenance and NLRP3 regulation. In summary, the NLRP3 inflammasome mediates crosstalk between HIV, mitochondrial dysfunction, and immune signaling, driving inflammation, neurodegeneration, and comorbidities in PLWH.

## 4 Purinergic receptors, ATP signaling, and inflammation

### 4.1 ATP-driven inflammasome activation and chronic inflammation

HIV infected cells show altered OxPhos activity with acute infection, which resembles increased ATP production. Latent infection is characterized by the upregulation of OxPhos and pannexin production (Freeman et al., 2023). OxPhos hyperactivation in HIV-infected cells is often coupled to pannexin-1 channel opening, enabling ATP efflux into the extracellular space. This ATP acts on purinergic receptors such as P2X1 and P2X7, altering ionic flux and triggering caspase activation and IL-1β processing. Experimental data from HIV-infected lymphoid explants and macrophage cultures support this pathway, but direct *in vivo* demonstration in PLWH is lacking. Notably, mitochondrial ROS can synergize with ATP signaling by sensitizing NLRP3 to purinergic cues, suggesting a coordinated stress-response axis rather than isolated pathways (Swartz et al., 2015). This upregulation of ATP production leads to inflammation in neighboring immune cells, bridging the gap between altered mitochondrial activity and increased inflammatory activity.

Crucially, in CD4 T cells, both OxPhos and pannexin-linked genes are upregulated, allowing ATP to rapidly leave the cell through pannexin1, a transmembrane ion channel (Freeman et al., 2023). This results in extracellular fluid rich in ATP, which then activates purinergic receptors. Purinergic receptors P2X1 and P2X7 are found in the cell membranes of CD4+ cells. They are cation channels, which, when bound to ATP, undergo a conformational change and allow cation flow into the cell. By transporting these cations in and out of the cell, P2X1 and P2X7 shift the charge of the cell by letting in Na^+^ and Ca²^+^. The change in charge causes potassium ions to rush out of the cell, triggering a chain of proinflammatory signaling. Ultimately, this activates caspase, an enzyme that cleaves the precursor form of IL-1β (pre-IL-1β) into the proinflammatory form, IL-1β (Peng et al., 2023). The altered mitochondrial landscape of HIV-infected cells can change the chemical landscape of the cell and surrounding cells, providing an ideal environment for proinflammatory molecule activation. It is important to note that excess extracellular ATP causes P2X1 to become overexposed and close. Nevertheless, purinergic receptors remain open for brief periods after prolonged exposure to ATP and trigger inflammation (Giniatullin and Nistri, 2013; Peng et al., 2023). This suggests that overactivation of P2X1 is not a self-repairing issue, and therapeutic intervention remains favorable.

### 4.2 CD39 regulation of extracellular ATP and implications for HIV persistence and immune cell dysfunction

CD39 and CD73 are two ectoenzymes that catalyze the ATP to adenosine reaction, thereby dampening immune activation. This reaction dampens inflammasome activation because the ATP required to activate purinergic receptors, which is upstream in inflammasome activation, is no longer available. Additionally, A2A signaling dampens proinflammatory signaling (Nikolova et al., 2011). In cells with active HIV infection, CD39 is upregulated, essentially turning off the ATP signaling for purinergic receptors. This favors the proliferation of the virus by diminishing the inflammatory response to it. With less inflammatory response, CD8+ T cells will not kill cells hosting the virus (by producing proinflammatory cytokines), and regulatory T cells will have reduced inflammatory response, providing the virus with ample replication grounds. This favors faster development of AIDS. In HIV-infected cells, CD73 is typically downregulated, so there is no conversion to adenosine, meaning there is no anti-inflammatory A2A signaling, and therefore CD39 is not a complete inhibitor of inflammation (Nikolova et al., 2011).

### 4.3 NF449 as a model purinergic antagonist impacting HIV entry and immunometabolic regulation, and potential novel therapies

Purinergic receptors also play a critical role in initial HIV infection. For HIV to bind to the host cell its receptors must be able to bind complementary host cell receptors. Recent research suggests that NF449 interferes with the viral membrane directly to inhibit viral fusion (Soare et al., 2020). At a benzene disulfonic acid group in NF449, there is overlap with the binding position of ATP, making the ATP bond with P2X1 non-functional (Qiang et al., 2025). NF449 is a large polar molecule derived from suramin, an antiparasitic drug, that binds to P2X1. The NF449 molecule however, is only approved for scientific use, according to manufacturers, and has a similar efficacy to AZT (in terms of viral fusion inhibition) (Soare et al., 2020). NF449 has intense effects on other proteins not targeted in purinergic receptor therapy and has low bioavailability (Qiang et al., 2025), making it ill-suited for use as a drug therapy. However, compounds with similar mechanisms of action to NF449 could potentially serve as drugs for PWLH with chronic inflammation.

## 5 Clinical relevance: mito-immune dysfunction in HIV-associated comorbidities

### 5.1 HIV-associated neurocognitive disorders

The mechanistic pathways described earlier converge with striking consequence in the central nervous system. Persistent mitochondrial dysfunction in microglia, astrocytes, and infiltrating immune cells sustains a state of chronic neuroinflammation in people living with HIV (Datta et al., 2019; Velasquez et al., 2019; Cheney et al., 2020; Hileman et al., 2021). This inflammatory milieu correlates with the clinical spectrum of HAND, from asymptomatic neurocognitive impairment to severe dementia. Elevated concentrations of IL-1β, soluble CD14, and neurofilament light chain in cerebrospinal fluid provide measurable biomarkers of this ongoing injury. Even in individuals with complete virologic suppression, cognitive decline may progress, reflecting the resilience of CNS reservoirs and the self-perpetuating nature of mitochondrial and inflammasome-driven pathology. Antiretroviral regimens, particularly those with documented mitochondrial toxicity, can compound this burden, amplifying oxidative stress and accelerating synaptic and neuronal loss (Rodriguez et al., 2024).

### 5.2 Cardiometabolic diseases

A parallel narrative unfolds within the cardiovascular system. Mitochondrial injury in endothelial cells and cardiomyocytes fosters oxidative stress, disrupts nitric oxide signaling, and impairs vascular homeostasis (Parikh et al., 2015; Teer and Essop, 2018). These changes precede and predict clinical events such as myocardial infarction and stroke in ART-treated cohorts. Large epidemiologic studies consistently demonstrate a higher incidence of cardiovascular disease in people living with HIV than in matched uninfected populations, even after rigorous adjustment for traditional risk factors. ART-associated dyslipidemia and adipose tissue dysfunction further intensify metabolic strain (Murata et al., 2000; Murata et al., 2002; Maagaard and Kvale, 2009), creating an environment in which vascular injury and systemic inflammation are mutually reinforcing. This confluence of virologic, immunologic, and metabolic forces produces a cardiovascular phenotype that is unique to the HIV population and not entirely mitigated by conventional preventive strategies.

### 5.3 Cellular senescence and accelerated aging

Beyond neurocognitive and cardiometabolic complications PLWH exhibit features of accelerated biological aging including increased senescence-associated T cells and premature multimorbidity. Mitochondrial dysfunction contributes directly to this process: persistent ROS production, mtDNA instability, and chronic inflammasome activation promote DNA damage and reinforce senescence-associated inflammatory phenotypes. These changes sustain systemic inflammation and immunosenescence, linking mitochondrial injury to frailty, osteoporosis and early cardiovascular and neurocognitive decline (Hileman et al., 2021; Ambikan et al., 2022). Recognizing accelerated aging as a consequence of mito-immune dysfunction underscores the need for therapies that preserve mitochondrial health and limit senescence driven inflammation.

### 5.4 Therapeutic interventions targeting the mito-immune axis

The translation of mechanistic insight into therapeutic innovation is both a challenge and an opportunity. Agents such as metformin, already widely used in metabolic disorders, have shown the capacity to restore OxPhos and temper inflammasome activation (Guo et al., 2021; Pulipaka et al., 2023; Rezaei et al., 2024). Mitochondria-targeted antioxidants, including naringin, and pleiotropic agents such as statins offer the potential to attenuate oxidative injury while modulating lipid metabolism (Tripathy and Mitra, 2010; Kallianpur et al., 2020; Rezaei et al., 2024). Experimental purinergic receptor antagonists such as NF449 and suramin illustrate how interference with ATP-driven inflammatory signaling might be coupled with antiviral effect (Swartz et al., 2015; Soare et al., 2020). These candidates point toward an emerging paradigm in which antiretroviral therapy is complemented by agents that preserve mitochondrial integrity and recalibrate immune function. Several direct NLRP3 inhibitors are under active investigation, including MCC950, dapansutrile, and related sulfonylurea derivatives, which block inflammasome assembly and downstream IL-1β release. Although not yet tested extensively in PLWH, these compounds have shown efficacy in preclinical models of inflammatory and metabolic disease, highlighting their potential to attenuate chronic HIV-associated inflammation (Bracey et al., 2013; Kelley et al., 2019). Emerging drug delivery strategies, including nanoparticle-based formulations, are being explored to improve mitochondrial targeting and antioxidant bioavailability. Such approaches may enhance the efficacy of compounds like metformin, esculetin, and mitochondria-targeted antioxidants already under investigation (Pulipaka et al., 2023; Rezaei et al., 2024).

## 6 Discussion

### 6.1 Future directions

The next phase of research must integrate mechanistic precision with clinical ambition. Longitudinal studies employing advanced imaging and multi-omic profiling can chart the trajectory of mitochondrial injury in diverse tissues over the course of treated infection (Tripathy and Mitra, 2010; Kallianpur et al., 2020). Therapeutic development should focus on molecules with the capacity to penetrate sanctuary sites, modulate mitochondrial metabolism, and suppress inflammasome activity without impairing host defense (Pulipaka et al., 2023; Rezaei et al., 2024). Future clinical trials should incorporate composite endpoints that extend beyond viral suppression to include restoration of mitochondrial function (e.g., respirometry in sorted immune cells), reduction of inflammasome biomarkers (plasma IL-1β, extracellular ATP, cell-free mtDNA), and prevention of end-organ disease. Candidate interventions—ranging from mitochondria-targeted antioxidants (MitoQ, SkQ1) to selective NLRP3 inhibitors (dapansutrile) and purinergic antagonists—should be evaluated for both efficacy and ability to penetrate sanctuary sites such as the CNS. The intersection of mitochondrial stress and immunosenescence in aging PLWH warrants particular focus, as this population is disproportionately affected by multimorbidity (Parikh et al., 2015; Teer and Essop, 2018).

### 6.2 Conclusions

Mitochondria have emerged from the periphery of HIV research to occupy a central position in the understanding of persistent immune activation and its clinical sequelae (Valle-Casuso et al., 2019; Guo et al., 2021). Their role extends beyond bioenergetics into the orchestration of inflammatory responses and the shaping of tissue resilience. Therapeutic strategies that protect and restore mitochondrial function hold the promise of transforming HIV care in the era of effective antiretroviral therapy (Swartz et al., 2015; Soare et al., 2020; Rezaei et al., 2024). By addressing the root causes of chronic inflammation and immune dysfunction, these approaches offer the possibility of extending both the health span and quality of life for people living with HIV.
